# Report on the Sanitary Condition of the City of Chicago

**Published:** 1861-10

**Authors:** N. S. Davis

**Affiliations:** Member of the Sanitary Committee


### ARTICLE IV.

### REPORT ON THE SANITARY CONDITION OF THE CITY OF CHICAGO.

Prepared for the Regular Meeting of the Chicago Medical Society, held Sept. 20th, 1861.

By N. S. DAVIS, M. D., Member of the Sanitary Com.

Since the appointment of your committee, at the annual meeting of the Society in April last, the general health of the community has been good. The city has been visited by no epidemics; and the prevalence of ordinary sporadic and endemic cases of disease, has been less than the average of the last six years. This is not owing to any superior cleanliness of streets or other sanitary measures adopted by the city government, but is undoubtedly owing to the small amount of hot, sultry weather during the past summer. From memoranda made during a series of years, it appears that in this locality, we usually have the commencement of high summer temperature between the twentieth of June and the tenth of July, and the commencement of the ordinary endemic diarrhœa, cholera morbus, and cholera infantum, dates at the same period of time. This year, however, we had no high summer temperature until the very last days of July and the first week in August. Then we had eight or ten days of oppressively hot weather, with a prevalence of south and south-west winds. This was succeeded by a severe storm from the north and north-east, and the continuance of a comparatively low summer temperature up to the present time. From the records of mortality, as well as from present observations, it appears that the annual prevalence of bowel affections, especially among children, usually dates its commencement with the first week of high temperature during the last of June, or first of July, and they continue more or less severe until past the middle of September. This year, however, according to my own observations, attacks of diarrhœa and cholera morbus, whether in children or adults, were much less frequent than usual, during all the month of July. The correctness of this is sustained by the diminished mortality of that month, as compared with previous years. Thus, the mortality of July, 1860, was 288, while that of July, 1861, was only 239. Another feature worthy of careful attention is, that while the number of cases of diarrhœa and vomiting occurring during July was much less than usual, there was a greater tendency to take on the form of dysentery. So manifest was this tendency, that in almost every case which had continued three days or more, the skin was dry, the pulse accelerated, and the intestinal discharges mixed with mucus, and not unfrequently streaks of blood. On the accession of the hot, sultry weather during the first week of August, attacks of diarrhœa and cholera morbus, especially among young children, became rapidly increased, both in frequency and severity; and in a few cases, symptoms of collapse were early manifested. During that period, several cases of "*sun-stroke*" were reported in the papers, some of them fatal. None came under my observation, however, and from some inquiries, I am inclined to think that most of those reported in the daily papers, were affected more by *whisky* than the sun.

During the brief period of high temperature alluded to, I saw three cases in adults, strongly resembling spasmodic cholera. The violent vomiting and purging of serous fluid, was accompanied by cramps in the muscles of the extremities; husky voice; blueness and corrugation of the skin; sunken eyes; full pulse, and coldness of surface. Only one of these cases terminated fatally. Since the storm that abruptly terminated the brief period of high temperature in the early part of August, intestinal affections have assumed much the same character as they exhibited during the middle and latter part of July; that is, the *number* of attacks has been less, with a greater tendency to dysentery. During the last three weeks primary attacks of diarrhœa or vomiting in children have been rare, while cases of ordinary dysentery among adults have been more frequent. Most of the cases of dysentery have been mild and easily controled by ordinary remedies. Within the circle of my observation, the summer has been unusually exempt from fevers of any kind, having hardly met with a well-marked case of continued fever previous to the first of September. Since that date, cases of typhoid or enteric fever have been gradually increasing in frequency. They have been mostly mild and devoid of unusual features. Within the last two weeks I have met with several cases of well-marked typhus. All but one, however, belonged to the same family. This family had recently moved into the city from a miserably constructed shanty, in which they had been living, on the open prairie during all the summer. The mother and five children were all prostrate with the disease. The father and the youngest child, a nursing infant, were the only members of the family exempt. Most of them had been two weeks sick, without any medical treatment, when I was called to see them. By the internal use of chlorate of potassa, and a fair supply of milk and animal broth, three of them have slowly recovered. Two of the children still remain in a feeble state from the continuance of a moderate diarrhœa, after apparent convalescence from the general fever.

During the week of very hot weather near the first of August, I was called to an unusual number of cases of convulsions in children. I also noticed during the same time, a peculiar manifestation of nervous and cerebral symptoms in children affected with diarrhœa. In some, who had been much prostrated by the excessive evacuations, the functions of the brain became exceedingly depressed, especially during the afternoon of each day. Between one and three o'clock, p. m., they would fall into a partially comatose condition, the face pale; the eyes partially closed; the chin dropped; the respiration slow and sometimes sighing; the pulse feeble; and the pupils moderately dilated. From this state, it was difficult to arouse them, for five or six hours. In a few instances, instead of this dormant, or partially comatose condition, there was great restlessness; a constant rolling of the head; tossing of the limbs; moaning, etc., with dilated pupils; but *sunken fontanelle.* These symptoms were evidently owing to cerebral exhaustion, produced by the combined influence of excessive evacuations and an unusually high temperature. They were best relieved by tannate of quinine, chlorate of potassa, and diffusible stimuli, aided by such anodynes as were necessary to control the intestinal evacuations.

In addition to the cases of diphtheria reported verbally at the meeting of the Society in July, I have met with four others, the last of which occurred only a few days since. They presented nothing worthy of notice, except that one of the cases occurring in the person of a young lady, was followed by paralysis of the fauces, rendering deglutition awkward; causing liquids sometimes to regurgitate through the nostrils, and giving to the voice a very unpleasant *nasal* sound. The diphtheritic inflammation was only of the average degree of severity, and was accompanied by only a moderate degree of swelling and ulceration. My attention was not called to the paralyzed condition of the fauces until some two weeks after all *appearances* of inflammation had disappeared from the neck and throat. There was a moderate degree of general debility, but the patient was more annoyed by the alteration of her voice than anything else. Under the influence of the following prescription, coupled with a plain but nutritious diet, and moderate exercise in the open air, the paralysis entirely disappeared in about two weeks:

℞ Strychnia, - - - grj.

Nitric acid, - - - ʒj.

Water, - - - - ℥ij.

Mix, and take a teaspoonful in sweetened water, before each meal. I have directed the same treatment in five or six cases of paralysis following diphtheria, and with like success. In closing this report, I would call the attention of the Society to the fact, that reports of deaths are frequently made, in which the cause of death is said to be "teething."

Is there any such disease as "teething?" If so, will some member of the Society inform us of its symptoms and pathology?

